# Field study to investigate the effectiveness and safety of a novel orally administered combination drug product containing milbemycin oxime and lotilaner (Credelio^®^ Plus) against natural intestinal nematode infections in dogs presented as veterinary patients in Europe

**DOI:** 10.1186/s13071-021-04766-7

**Published:** 2021-05-17

**Authors:** Brad Hayes, Scott Wiseman, Daniel E. Snyder

**Affiliations:** 1 Elanco Animal Health, Bartley Way, Hook, Hampshire RG27 9XA UK; 2 Daniel E. Snyder DVM PhD Consulting, LLC, Indianapolis, IN 46229 USA

**Keywords:** *Ancylostoma caninum*, Credelio Plus, Dog, Effectiveness, Lotilaner, Milbemycin oxime, Oral, T*oxascaris leonina*, *Toxocara canis*, *Trichuris vulpis*, Veterinary patients

## Abstract

**Background:**

A randomised, blinded, positive controlled, multicentre, Good Clinical Practice-compliant, pivotal field study was conducted to evaluate the effectiveness and safety of a new combination of lotilaner + milbemycin oxime tablets (Credelio^®^ Plus; Elanco Animal Health) administered orally to client-owned dogs naturally infected with intestinal nematodes.

**Methods:**

Client-owned dogs presenting to veterinary clinics from households in France, Hungary and Germany were screened for intestinal nematodes. Dogs with an initial positive faecal egg count that was subsequently confirmed with a follow-up faecal examination to demonstrate the presence of naturally occurring mixed or mono-infections with *Toxocara canis*,* Toxascaris leonina*,* Trichuris vulpis* or *Ancylostoma caninum* were enrolled on Day 0 into the study. Households were randomised in an approximately 2:1 ratio to receive either an investigational product (IP; Credelio Plus tablets) or control product (CP; Nexgard Spectra^®^ tablets) as treatment. Dogs were administered the IP (*n* = 278) or CP (*n* = 117) once on Day 0 at a dose rate of 0.75–1.56 mg/kg bodyweight milbemycin oxime and 20.0–41.5 mg/kg bodyweight lotilaner (IP) or as recommended (CP). Effectiveness of the IP and CP treatments was based on the post-treatment reduction in geometric mean faecal egg counts on Day 8 (range Day 7–10) after treatment as compared to their pre-treatment nematode faecal egg counts.

**Results:**

Geometric mean (GM) faecal egg counts for *T. canis*, *A caninum* and *T. vulpis* were reduced by ≥ 97.2% in the Credelio Plus group and  by ≥ 95.3% in the afoxolaner + milbemycin oxime group. There were insufficient data to calculate a percentage reduction in GM faecal egg counts between Day 0 and Day 8 for *T. leonina* due to low prevalence. Credelio Plus was well tolerated in this field study. Of the 355 total doses administered, 82.3% were accepted free choice in the IP group compared to 80.8% in the CP group.

**Conclusions:**

This study demonstrated effectiveness (≥ 97.2% reduction), safety and tablet acceptance of a combination of milbemycin oxime and lotilaner (Credelio Plus) administered orally to dogs with natural intestinal infections of *T. canis, A. caninum and T. vulpis.*
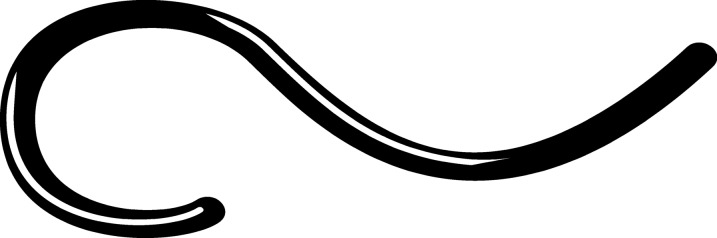

## Background

Intestinal nematode infections are commonly diagnosed in dogs from all parts of the world, including Europe [1–4]. Of these intestinal nematodes, *Ancylostoma caninum* and *Toxocara canis* are considered to be two of the most important and commonly reported helminth parasites of dogs [5, 6]. Infected dogs can play an important role in the transmission of these two common zoonotic nematodes by excreting eggs directly into the environment where humans and other dogs can be exposed. The veterinary and public health aspects of hookworm and *T. canis* infections in dogs are well established [5, 7, 8]. In Europe, *A. caninum* is one of two hookworm species routinely found in dogs; however, it is reported to be found predominantly in dogs located in southern parts of Europe [9].

Endectocidal combination products or anthelmintics with a broad spectrum of activity can provide the pet owner and veterinarian with the means to treat dogs that are concurrently infested or infected with multiple parasite types. The effectiveness of milbemycin oxime (MO) in combination with other drug substances administered to dogs naturally infected with different species of adult intestinal nematodes has been previously demonstrated in laboratory dose confirmation studies [10]. It has also been shown that a minimum dose of 0.75 mg/kg of MO will effectively treat larval and immature adult stages of *T. canis* and *A. caninum* [11].

The two drug substances of the fixed combination product assessed in this field study are lotilaner, which has activity against several ectoparasites (i.e. fleas, ticks, mites), and MO, which has activity against a number of clinically important intestinal nematode species in dogs [10, 12, 12–25]. Client-owned dogs attending veterinary clinics in France, Germany and Hungary were enrolled in this field study to assess the fixed combination of MO + lotilaner for the treatment of *T. canis*,* Toxascaris leonina*,* Trichuris vulpis* and *A. caninum* infections. It is intended that this drug product will be used monthly where necessary. Demonstration of the existence of concurrent infestations/infections was performed as part of the justification of the combination of these two drug substances for dogs enrolled in this study. Data on the presence or absence of fleas and/or ticks prior to treatment were therefore also collected although efficacy against these parasites was not evaluated in this study. The primary objectives of this field study were to evaluate the effectiveness and safety of this novel MO + lotilaner combination drug product administered as an oral chewable tablet as compared to an authorised control product (CP) for the treatment of naturally occurring intestinal nematode infections diagnosed in client-owned dogs in Europe presenting as veterinary patients under end-use clinical field conditions. A secondary objective, acceptance of the tablet by enrolled dogs, was also evaluated.

## Methods

The use of the MO + lotilaner combination investigational product (IP) in this study was intended to evaluate a proposed commercial formulation and dose regimen for use in dogs under end-user conditions for the intended European intestinal nematode label indications (Credelio^®^ Plus; Elanco Animal Health, Greenfield, IN, USA). As such, this was a pivotal randomised, blinded, positive controlled, multicentre, Good Clinical Practice (GCP)-compliant, European field study with the aim to evaluate the effectiveness and safety of a combination of MO + lotilaner chewable tablets (IP) administered orally to dogs. Dogs were enrolled from a total of 38 veterinary clinical sites located in Germany (*n* = 14), Hungary (*n* = 15) and France (*n* = 9). Trial clearances for the conduct of this field study in client-owned animals for each of these countries were approved prior to enrolling the dogs. Data from all countries were pooled. To obtain the required numbers of dogs for this study, it was necessary to screen 2848 dogs. This field study was conducted in accordance with the principles of GCP as established in the VICH guidelines GL9, Good Clinical Practice (June 2000); The Efficacy Requirements for Anthelmintics: Overall Guidelines (VICH GL7); The World Association for the Advancement of Veterinary Parasitology (WAAVP) guidelines for evaluating the efficacy of anthelmintics for dog and cats; VICH GLl9 Effectiveness of Anthelmintic; Overall Guidelines: Specific Recommendation for Canines (June 2001); and the Guideline on the demonstration of palatability of veterinary medicinal products EMNCVMP/EWP/206024/2011 [26–30]. Personnel at the different study sites involved in making assessments of intestinal nematode effectiveness and safety and personnel at the designated laboratories performing faecal examinations for nematode eggs were masked to treatment assignments. Day 0 was defined as the day the treatment was administered to each enrolled dog.

### Study design

The study was a multi-site clinical study involving client-owned dogs under field conditions. Dogs presenting to each veterinary clinic were screened from Day-14 to Day-7 by collecting a fresh 3-g faecal sample to be processed using a qualitative faecal examination method (methods described below). A positive result at screening (i.e. > 0 parasite eggs from *Toxocara canis*, *Toxacaris leonina*, *Trichuris vulpis* or *Ancylostoma caninum*) enabled the dog to proceed to Day 0 faecal sampling, enrollment and dosing. A dog that was initially positive for faecal eggs and was administered treatment but had zero eggs on the Day 0 faecal egg count (FEC) or was diagnosed with a mono-specific *Uncinaria* infection was excluded from the efficacy evaluable population but was included in the safety evaluable population. To be enrolled in the study, on Day 0 the owner was required to complete and sign an owner Consent Form and to provide the dog’s prior and current medical history. Dogs considered for enrollment had to be at least 8 weeks of age and weigh ≥ 2.0 kg on Day 0. At the clinic visit on Day 0, each dog with a confirmed positive screening faecal sample was weighed, another faecal sample was obtained and the dog was additionally given a physical examination. During the physical examination, the dog was also examined for the presence of adult fleas, flea frass and/or attached ticks. If fleas or ticks were present, this was recorded in the study documents.

Single- and multi-dog (maximum of 4 dogs per household) households were eligible for participation. For multi-dog households, when more than one dog within a household was positive for faecal eggs at screening, all positive dogs in the household received the same treatment. The effectiveness of the IP or CP was evaluated in each enrolled treated dog with a positive Day 0 FEC. All households were randomised to one of two groups of either IP or CP treatment. Analysis of safety data included all dogs receiving either IP or CP on Day 0 of the study. Oral tablet acceptability was evaluated in the product safety population from acceptability data associated with the method of treatment.

Dogs of any breed and sex, reproductively neutered or intact (non-pregnant and non-lactating if female) and not intended for breeding during the study were eligible for enrolment. Other animal eligibility criteria included: the dog was of a suitable temperament (not fractious); the dog was owned by a client, not an investigator or the clinic’s veterinarian(s), staff, or relatives thereof; if housed primarily outdoors, each dog in the household was maintained in a controlled home/yard environment that allowed for observations required by the study; the dog was generally healthy based on physical examination; the dog was free of a serious disease that would interfere with the objectives of the study.

### Randomisation

Dogs were allocated to treatment using a randomisation produced by an electronic data capture system (Prelude Dynamics, Austin, TX, USA). The randomisation was specific to the site and was unique for single- and multi-dog households. All positive dogs in the same household received the same treatment. Assignment of case numbers to dogs was separate from allocation to treatment. Case numbers were assigned to dogs by order of enrolment. Only those dogs with a positive FEC which met all of the inclusion criteria and for which none of the exclusion criteria applied were allocated to treatment. Dogs were blocked by consecutive order of enrolment in the study. Each site's random allocation tables provided for enrolment in a 2:1 ratio for the IP and CP groups, respectively. Within single-dog households, sets of three dogs with positive FEC were allocated within each block (two to IP and one to CP). For multi-dog households, when more than one dog within a household was positive for faecal eggs at screening, all positive dogs in the household received the same treatment in order to mitigate the risk of treatment with the wrong product. Dogs from multi-dog households had to be dosed on the same Day 0 with the same treatment. For multi-dog households, each block comprised three households, two of which were allocated to IP and one to CP. If the household consisted of multiple dogs but only one was positive for faecal eggs at screening, the dog was still allocated from the multiple dog randomisation list.

### Treatment

Dogs in each household with a positive screening FEC were administered IP or CP once on Day 0. A designated unmasked dispenser at each veterinary clinic was solely responsible for dispensing of the IP and CP and giving and reviewing product administration instructions to the owners. The IP chewable tablets were supplied in five different strengths to provide the targeted dose range of the flavored tablet for the IP and a unit dose reflecting the intended oral treatment at approximately 20–41.5 mg lotilaner per kg bodyweight and approximately 0.75–1.56 mg MO per kg bodyweight. The CP, marketed as Nexgard Spectra^®^ (afoxolaner + MO; Boehringer Ingelheim, Ingelheim am Rhein, Germany), was dispensed per label directions for the European market (dose rate: 2.50–5.36 mg/kg bodyweight of afoxolaner and 0.50–1.07 mg/kg bodyweight of MO).

Each owner was instructed on storage and administration of the assigned product in the home environment while at the veterinary clinic. At home, each owner administered the tablets and confirmed and documented product consumption. Each product was given under the following conditions to ensure maximum product effectiveness. First, the IP was to be administered to the dog under the fed condition (last meal taken approximately 30 min prior to treatment); if the dog had not been fed approximately 30 min prior to treatment, or in case of doubt, food was to be offered to the dog (the targeted amount was one third of the daily ration) prior to treatment. Secondly, the CP tablets were orally administered as per the manufacturers label instructions, which did not require that the dogs be fed prior to dosing. Thirdly, the owners also recorded the way the tablets were consumed to measure tablet acceptance. Owners were first asked to offer the tablet in an empty bowl for approximately 1 min. If after this time the tablet had not been consumed, then they were offered by hand for approximately another minute. If after both these offerings the dog had still not fully consumed the tablet, then the tablet was either offered in food or the dog was manually dosed. Finally, the owner returned with all treated dogs in each household to the clinic on Day 8 (range Day 7–10).

### Faecal egg counts

Fresh faecal samples were obtained from each dog presented at the veterinary clinic and placed in containers supplied as part of the study. Each faecal sample was from a recently voided sample or obtained directly (rectally) from each dog (minimum 3 g wherever possible) at each of the scheduled study time points. All faecal samples were refrigerated at each veterinary clinic until they were shipped to the testing laboratory. All faecal samples (screening, Day 0 and Day 8) were shipped overnight in insulated shipping containers with ice packs to the Charles River Laboratories Ireland Ltd parasitology laboratory (Ballina, County Mayo, Ireland). When samples were received at the laboratory, they were identified by their unique identification number (screening or case number) and the date of sampling. Faecal samples were stored refrigerated upon receipt until analysis took place.

Nematode infections were assessed at the designated laboratory by suitably qualified and trained personnel who were blinded to the allocation of animals to treatment. Faecal samples for assessment of *T. canis*,* T. leonina*,* T. vulpis* or *A. caninum* (and, if present, *U. stenocephala*) infections based on known morphological characteristics of eggs for these nematode parasites taken from dogs 7–14 days prior to Day 0, on Day 0 prior to treatment and then on Day 8 (range Day 7–10) after treatment [31]. The method used to determine the presence of (qualitative for screening) or the quantitative number of *T. canis*,* T. leonina*,* T. vulpis* or *A. caninum* eggs in each faecal sample was a double centrifugation method with a flotation solution consisting of a saturated sugar solution (specific gravity ≥ 1.27) [31]. This method has a sensitivity of 1 egg/g of faeces. Every parasite egg visualised was identified to species under the microscope, counted and recorded as the number of parasite eggs by species per gram of faeces. 

If hookworm eggs were found in the screening sample, an additional analysis was performed at the time of the FEC in order to ascertain whether the eggs were likely to be those of *Ancylostoma* or *Uncinaria* based on the size of the eggs present [31]. The testing laboratory assessed hookworm eggs seen during the screening examination based on the published size of morulated eggs of *A. caninum* and *U. stenocephala* in which they were differentiated microscopically based on their length, with *A. caninum* usually measuring < 79 μm and *U. stenocephala* measuring < 92 μm [31]. A positive result at screening (i.e. > 0 parasite eggs from *T. canis*,* T. leonina*,* T. vulpis* or *A. caninum* identified) enabled the dog to proceed to Day 0 sampling and dosing. A negative screening result (i.e. only other parasite eggs present, such as those of *U. stenocephala*, or no parasite eggs identified) meant the dog was not enrolled in the study.

### Safety assessments

The safety assessment was performed for all dogs that had received a treatment, either IP or CP, within each household by the pet owner. During the study period from the Day 0 treatment to study completion on the final study visit on Day 8 (range Day 7–10), study dogs treated with the IP or CP were observed daily by the owner in their household, including on the day of dosing. Each veterinary clinic was trained on monitoring and reporting adverse events (AEs) and additionally instructed the owners on the importance of AE monitoring. Physical examinations were performed at the final study visit. Owners were requested to inform the investigator whenever they observed any abnormalities in their dog(s). All observed or reported AEs were recorded. Any other medications given to a study dog during the course of the study, except the study treatments, were recorded.

### Statistical methodology/biometrics

Each treated dog in each household with a positive FEC on Day 0 was considered to be the experimental unit for nematode effectiveness assessments for the IP and CP groups. The safety assessment was performed for all dogs that received a treatment with either IP or CP. The 5% level of significance (*P* < 0.05 for two-sided tests) was used in all statistical testing. All statistical analyses used the statistical package SAS version 9.4 (SAS Institute, Cary, NC, USA). Demographics, enrolment and population, study completion/termination, dosing, tablet acceptance, baseline nematode infection and concurrent treatment usage data were summarised. The effectiveness of each treatment was declared when, based on geometric means (GM), the reduction in egg count exceeded 90% and was statistically significant.

The primary measure of efficacy was the percentage reduction in GM FEC between the pre-treatment Day 0 sample and the post-treatment (Day 8) sample. This was performed separately for each of the four parasites of interest in this study (*T. canis*,* T. leonina*,* T. vulpis* and *A. caninum*). For each of the individual nematode species, a minimum of two IP dogs and a minimum of two CP dogs were required to conduct statistical analyses. A secondary efficacy parameter was the reduction in total infection (total infection is defined as the sum of the egg counts from all 4 parasite species of interest enumerated). A natural logarithmic transformation (ln[count + *I*]) was applied to the pre-treatment and post-treatment counts for each individual animal to account for the asymmetric, skewed nature of these data, to stabilise the variance and to allow for zero counts. The log-transformed egg counts were analysed using repeated-measures mixed model methodology. Treatment group, time (pre- or post-treatment) and time-by-treatment group were fixed effects in the model. The site-by-treatment interaction and household within site-by-treatment were fitted as random effects. A dog within a household was also fitted as a random (repeated measures) effect to account for the correlation between observations on the same dog and between dogs within the same household. Different covariance structures were investigated to model the correlation between observations on the same dog/household, with the structure in the final model being selected based on the lowest Akaike Information Criterion value. A separate model was fitted for each of the primary and secondary efficacy variables.

The percentage reduction in FEC were calculated within the treatment group using back-transformed model least-square (LS) means, obtained from the statistical models, with the following equation: [(*C* − *T*)/*C*] × 100, where *C* is the LS mean pre-treatment FEC and *T* is the LS mean post-treatment FEC.

For each of the primary and secondary variables, the proportion of dogs successfully treated was also calculated; success in an individual dog was defined as a reduction of at least 90% in FEC post-treatment compared to the pre-treatment FEC. The proportion of successfully treated dogs was also compared between treatments using a standard non-inferiority assessment with a 15% equivalence margin. Non-inferiority between groups was declared when the lower limit of the 95% confidence interval for the difference in proportion of successful treatments (IP – CP) was greater than − 15%.

## Results

### Dog demographics: effectiveness evaluable population

Descriptive statistics on dogs enrolled on Day 0 in the effectiveness evaluable population are summarised in Table 1.

**Table 1:** Descriptive statistics for signalment and demographics of dogs enrolled on Day 0 as veterinary patients and included in the effectiveness evaluable population

Demographics of study dogs	European nematode field study	
	IP: Credelio Plus (*n* = 278)	CP: Afoxolaner + MO (*n* = 117)
Purebred, *n* (%)	160 (57.6)	60 (51.3)
Mixed breed, *n* (%)	118 (42.4)	57 (48.7)
Age, mean (months)	58.8	51.8
Age, range (months)	1.9–180.0	2.3–156
Bodyweight, mean (kg)	21.3	22.7
Bodyweight, range (kg)	3.5–53.3	2.8–70.0
Male, *n* (%)	143 (51.4)	67 (57.3)
Female, *n* (%)	135 (48.6)	50 (42.7)
Coat length, *n* (%)		
Short	126 (45.3)	52 (44.4)
Medium	123 (44.2)	56 (47.9)
Long	29 (10.4)	9 (7.7)
Number of dogs in each country (%)		
France	130 (46.8)	52 (44.4)
Germany	26 (9.4)	9 (7.7)
Hungary	122 (43.9)	56 (47.9)
Sleeping location, *n* (%)		
Inside	75 (27.0)	27 (23.1)
Outside	203 (73.0)	90 (76.9)
Presence of fleas or ticks, *n* (%)		
No	182 (65.5)	79 (67.5)
Yes	96 (34.5)	38 (32.5)

*IP* Investigational product; combination tablets of lotilaner + milbemycin oxime (MO), marketed as Credelio Plus (Elanco Animal Health, Greenfield, IN, USA);* CP* control product; combination tablets of afoxolaner + MO, marketed as Nexgard Spectra (Boehringer Ingelheim, Ingelheim am Rhein, Germany)

### Demographics and evaluable effectiveness nematode population

In order to obtain the required number of dogs positive for the nematodes of interest for the study, it was necessary to screen 2848 dogs. Of these dogs, 395 were enrolled in the nematode effectiveness evaluable population (278 dogs to the IP group and 117 dogs to the CP group; Table 1). The majority of the cases excluded (*n* = 111) from the efficacy evaluable population for the IP and CP groups (and thus the study) did not have a confirmed infection of the target species on Day 0 (*n* = 83) or had a monospecific infection with *Uncinaria* (*n* = 12). The reasons for dogs becoming negative for FEC from the screening examination up to the Day 0 examination is not known since the sensitivity of the egg count method that was used was 1 egg/g faeces. At the qualitative FEC screening exam, of the 111 excluded cases on Day 0, 73 of the 83 dogs had what was categorised as having few eggs of the target species. Additionally, 12 dogs on Day 0 had only mono-specific infections with *Uncinaria* and thus were excluded. The remaining cases excluded from the study were due to protocol non-compliance. As seen in Table 1, demographic data were similar between both groups.

### Safety

During the course of the post-treatment study period, all abnormal events, regardless of their causality, duration or severity were recorded by the pet owner for each enrolled dog that received an oral dose of either the IP or CP. Regarding the safety assessment aspect of this study, six AEs were reported for five of the 355 dosed dogs in the IP group (1.4%), three of which were diarrhoea, two were hepato-biliary disorders and one was lethargy. From the CP group, five AEs were reported for four of the 151 dosed dogs (2.6%), two of which were digestive tract disorders (diarrhoea; emesis), two were a skin disorder (alopecia; desquamation) and one was lethargy. The study site investigator assessed all owner-observed AEs as unrelated to the IP or CP treatments based on clinical examinations, history and timing of each event.

A number of concomitant medications were given to dogs in both the IP and CP groups. The most frequently administered concurrent treatments in both groups were vaccinations (24 dogs). Concurrent treatments used during the study included licensed animal drugs, such as antibiotics (12 dogs) and steroids (3 dogs), human drugs used off-label, alternative/herbal remedies, medicated shampoos or other topical treatments and prescription diets. These safety data, while only assessed over a short period of time after a single treatment, demonstrated that the IP and CP were well tolerated and used safely alone or with numerous concomitant treatments and vaccines routinely administered to dogs in veterinary medicine.

### Credelio Plus tablet acceptance

The IP was administered to 355 dogs in the safety population during the study, with 82.3% of these doses accepted free choice (e.g. taken from an empty bowl or from the hand) and 17.7% being administered by pilling (manually dosed). In comparision, in the CP group (151 dogs), 80.8% of these tablets were administered free choice and 19.2% were pilled. No doses were refused. A summary of tablet acceptance is presented in Table 2.

**Table 2:** Summary of tablet acceptance for dogs administered Credelio Plus or Nexgard Spectra tablets in the safety population

Treatment group	Number of dogs	Number of dogs consuming tabletfree choice (%)	Number of dogs manually dosed (pilled) (%)
Credelio Plus	355	292 (82.3%)	63 (17.7%)
Nexgard Spectra	151	122 (80.8%)	29 (19.2%)

### Nematode effectiveness

A total of 278 dogs treated with IP and 117 dogs treated with CP were assessed as part of the nematode effectiveness calculations across all nematode species assessed in this study (Table 3). The day 0 baseline GM FEC (range) for IP dogs infected with *T. canis*, * T. vulpis* and *A. caninum* were 16.4 (1–1276), 9.8 (1–797), and 11.2 (1–781), respectively. The day 0 baseline GM FEC (range) for CP dogs infected with *T. canis, T. vulpis* and *A. canimum* were 8.0 (1–574), 9.7 (1–683) and 9.9 (1–369), respectively. In this European field study, the GM FEC for *T. canis*, *A caninum* and *T. vulpis* were reduced by ≥ 97.2% in the Credelio Plus group. The GM FEC for these same parasites were reduced by ≥ 95.3% in the afoxolaner + MO (CP) group. There was insufficient data to calculate a percentage reduction in GM FEC between Day 0 and Day 8 for *T. leonina.* These results show that the effectiveness of Credelio Plus was non-inferior to that of NexGard Spectra. Mixed nematode infections were seen in 58 dogs in the IP group (20.9%) and in 22 dogs in the CP group (18.8%). The number of dogs successfully treated and the percentage success rate for the IP- and CP-treated dogs was 254 (91.4%) and 103 (88.0%), respectively, with successful treatment defined as at least a 90% reduction in FEC in an individual dog relative to Day 0 (Table 4).

**Table 3:** Effectiveness of a single oral dose of Credelio Plus against natural nematode infections in dogs presenting to European veterinary clinics

NNematode species	Sample^a^	Treatment group^b^	Number of dogs	Egg count per gram faeces			
				Geometric Mean (GM)	GM % reduction	Number of dogs with zero eggs (%)	Effectiveness *P*-value^c^
All nematode species	Pre-treatment	Credelio Plus Afoxolaner + MO	278 117	13.2 11.2	– –	– –	– –
	Post-treatment	Credelio Plus Afoxolaner + MO	278 117	0.3 0.3	97.6 97.0	245 (88.2%) 99 (84.6%)	< 0.0001 (*t*_524_ = 24.33) < 0.0001 (*t*_524_ = 14.65)
*Toxocara canis*	Pre-treatment	Credelio Plus Afoxolaner + MO	54 19	16.4 8.0	– –	– –	– –
	Post-treatment	Credelio Plus Afoxolaner + MO	54 19	0.5 0.4	97.2 95.3	48 (88.9%) 16 (84.2%)	< 0.0001 (*t*_79.3_ = 4.36) < 0.0001 (*t*_79.3_ = 9.70)
*Ancylostoma caninum*	Pre-treatment	Credelio Plus Afoxolaner + MO	109 42	11.2 9.9	– –	– –	– –
	Post-treatment	Credelio Plus Afoxolaner + MO	109 42	0.2 0.5	98.1 95.3	100 (91.7%) 35 (83.3%)	< 0.0001 (*t*_185_ = 9.28) < 0.0001 (*t*_185_ = 17.19)
*Trichuris vulpis*	Pre-treatment	Credelio Plus Afoxolaner + MO	176 80	9.8 9.7	– –	– –	– –
	Post-treatment	Credelio Plus Afoxolaner + MO	176 80	0.2 0.1	97.8 98.5	156 (88.6%) 73 (91.2%)	< 0.0001 (*t*_340_ = 12.79) < 0.0001 (*t*_340_ = 18.57)

*MO* milbemycin oxime

^a^Pre-treatment sample collected once on Day 0; post-treatment sample collected on Day 8 (range 7–10)

^b^Single oral administration of Credelio Plus on Day 0 to provide a dose range of 0.75–1.56 mg/kg bodyweight MO and 20.0–41.5 mg/kg bodyweight lotilaner. Single oral dose of Nexgard Spectra on Day 0 to provide a dose range of 2.50–5.36 mg/kg bodyweight of afoxolaner and 0.50–1.07 mg/kg bodyweight of MO

^c^Effectiveness *P-*value is the *P*-value for the within-treatment difference in least-square means

**Table 4:** Summary of success rate following a single oral dose of Credelio Plus against natural nematode infections in dogs presenting to European veterinary clinics

Nematode species	Treatment group^a^	Number of dogs	Success rate^b^		Difference in success rate	
			Successful dogs	Success rate (%)	Estimate	95% Confidence interval^c^
All nematode species	Credelio Plus Afoxolaner + MO	278 117	254 103	91.4 88.0	3.33 –	(− 3.41, 10.08) –
*T. canis*	Credelio Plus Afoxolaner + MO	54 19	48 16	88.9 84.2	4.68 –	(− 13.74, 23.09) –
*A. caninum*	Credelio Plus Afoxolaner + MO	109 42	103 38	94.5 90.5	4.02 –	(− 5.84, 13.88) –
*T. vulpis*	Credelio Plus Afoxolaner + MO	176 80	161 75	91.5 93.8	− 2.27 –	(− 8.99, 4.45) –

*MO* milbemycin oxime

^a^Single oral administration of Credelio Plus on Day 0 to provide a dose range of 0.75–1.56 mg/kg bodyweight MO and 20.0–41.5 mg/kg bodyweight lotilaner. Single oral dose of Nexgard Spectra on Day 0 to provide a dose range of 2.50–5.36 mg/kg bodyweight of afoxolaner and 0.50–1.07 mg/kg bodyweight of MO

^b^Successfully treated dog is one for whom the individual percentage reduction in egg counts at Day 8, relative to Day 0, is at least 90%

^c^Confidence interval for the difference (Credelio Plus – Nexgard Spectra) in proportions of two independent samples. Non-inferiority is accepted when the lower limit of the interval is greater than − 15

## Discussion

The objective of this study was to evaluate the effectiveness and safety of Credelio Plus (lotilaner + MO), administered once for the treatment of intestinal nematode species in naturally infected client-owned dogs under field conditions in Europe, compared to an authorised reference product (CP). The ectoparasiticidal component of the IP, lotilaner, is already registered in the European Union for the treatment of flea (*Ctenocephalides felis* and *C. canis*) and tick (*Rhipicephalus sanguineus*, *Ixodes ricinus*, *Ixodes hexagonus* and *Dermacentor reticulatus*) infestations in dogs (lotilaner; Credelio™ chewable tablets for dogs) [12–16, 18–25]. To extend the effectiveness of the product when dogs have been diagnosed with concurrent nematode infections and also ectoparasite infestations, lotilaner was combined with MO to form Credelio Plus, a combination therapy. In this nematode field study, dogs were enrolled with positive FEC and were also found to be co-infested with fleas and/or ticks (approx. 34% for both treatment groups); the results indicate that the routine use of a broad-spectrum endectocidal drug product like Credelio Plus can treat or control multiple endo- and ectoparasites in dogs when used by pet owners and veterinarians. A large number of dogs were required to be screened (*n* = 2848) in order to identify dogs positive (*n* = 395 in both IP and CP) for nematode eggs and the species of interest. This prevalence, which is similar to prevalences reported in dog faecal surveys previously conducted in Europe [3, 4, 6, 32, 33], demonstrates that intestinal nematode infections are common (approx. 14% in this study) in dogs in Europe.

 The zoonotic nematode species *T. canis*, the highly pathogenic *A. caninum*, along with *T. vulpis*, were commonly diagnosed in this field study, further demonstrating the need to routinely conduct faecal examinations and to treat client-owned dogs for these different intestinal nematode parasites. The higher prevalence of *T. vulpis* was surprising and differs from prevalences reported in other studies conducted in dogs in Europe. The prevalence of *T. leonina* was very low, with only one dog in the CP group being positive during the screening process. In this study, a number of both IP- and CP-treated dogs had Day 0 pre-treatment FEC numbers that were very high for the species identified (e.g. *T. canis* range of 1–1275 and 1–574 for IP and CP, respectively), in line with overdispersion of parasites in dog populations, but at the same time confirming the potentially high risk of environmental contamination by non-treated, infected dogs in home environments and those locations shared with other dogs [34, 35]. The zoonotic potential of * T. canis* and pathogenic disease caused by *A. caninum* are well documented, including Europe [5–8, 32, 33]. Based on seroprevalance data, human exposure to *T. canis* has also been reported in different regions of the world where the age-adjusted *Toxocara* seroprevalence was 13.9% [36, 37].

 The most common control measures for *T. canis* include regular and frequent anthelmintic treatment of dogs starting at an early age, education on and enforcement of laws for the disposal of canine faeces, dog legislation and personal hygiene [38]. It is well documented that *T. canis* eggs are very environmentally resistant and can survive well over most winters in temperate climates, thus reducing environmental contamination from infected dogs is critical. *Toxocara canis* can be routinely found in both juvenile and mature dogs [39], so the routine monitoring and treating of dogs of all age classes should be a routine practice. The post-treatment effectiveness of Credelio Plus against all of the canine intestinal nematode species assessed in this field study was ≥ 97.2%. This high level of effectiveness against these three common nematode species is similar to that reported in other laboratory and field studies which assessed MO in combination with other drug substances; however, in some of these cited studies a lower minimum dose of 0.5 mg/kg of MO was utilised [10, 40, 41]. Credelio Plus, as a broad-spectrum endectocide can be used by pet owners and veterinarians to effectively treat dogs with adult and immature intestinal nematode infections. Various scientific expert groups, such as the European Scientific Counsel Companion Animal Parasites (ESCCAP), recommend to pet owners and veterinarians to provide regular treatment and control of all intestinal nematodes of dogs [42]. In particular, where only *T*. *canis* is a concern, deworming at least four times a year is recommended if dogs and cats are housed outside or have access to the outdoors. ESCCAP further points out that the routine treatment and prevention of all worms depends upon legislation in individual countries, veterinary professionals taking local epidemiological circumstances into account, owner perception and individual risk assessments, such as hunting pets, previous lungworm exposure, raw meat diets etc. Deworming practices should therefore always be on the advice of a veterinary professional. The use of Credelio Plus is of particular importance for zoonotic species like *T. canis* and the zoonotic and pathogenic hookworm species *A. caninum* that are commonly found in dogs of all ages and throughout the year in prevalence studies conducted in different areas of the world, including in healthy, well-cared for dogs in Europe [3, 4, 32, 33, 39, 42–47]. There is surprisingly little information about the impact of re-treatment intervals on parasite burdens and environmental contamination. Current information suggests that annual or twice-yearly treatments do not have a significant impact on preventing patent intestinal nematode infections within a population of dogs, so a treatment frequency of at least 3–4 times per year is a general recommendation that may reduce faecal egg shedding and environmental contamination [42]. Monthly deworming treatments can treat existing patent infections of these different intestinal nematode parasites. One study showed that the prevalence of common nematode and cestode endoparasites declined significantly in a population of well-cared for dogs that were analysed, with the authors attributing the decline to the monthly use of broad-spectrum endectocides [48]. The MO minimum dose (0.75 mg/kg) in Credelio Plus has also been shown to effectively treat dogs with L4 larvae and immature adult stages of both *T. canis* and *A. caninum*, thus preventing the establishment of patent infections and stopping environmental contamination [11].

Safety was also assessed in this study in which 355 doses of the IP combination product were administered to dogs; however, this field study assessed only a single treatment on Day 0 and then only followed these animals for about 8 days to assess AEs. AEs documented during the study were consistent with abnormal observations seen globally by pet owners and routinely seen in any general dog population, such as diarrohea and emesis. The AE profile of Credelio Plus in this field study was similar to that seen with the CP, Nexgard Spectra. Lotilaner (Credelio™) as a standalone product administered as an oral ectoparasiticide has been reported to have a similar AE profile in dogs under field use and laboratory conditions, also including lethargy and diarrohea [19]. Abnormal health observations documented in this field study were not unexpected as the single components of the combination product have been commonly used and/or are well characterised in dogs. MO used alone or in combination with other oral parasiticides has been used safely for over 20 years for intestinal nematode control and heartworm and lungworm prevention in dogs; however, the dose range of MO in most of these products is from 0.5 to 1.0 mg/kg bodyweight, while Credelio Plus has a MO dose range of 0.75 to 1.56 mg/kg bodyweight [49]. Thus, there is some overlap in the dose ranges of MO in these marketed drug products. Lotilaner (Credelio™) as a standalone product administered as an oral ectoparasiticide has demonstrated safety for dogs under both field use and laboratory conditions [13–26]. Of the 355 doses of the IP combination tablets offered to dogs in this field study, 82.3% of doses were accepted free choice as compared to the CP that had a free choice acceptance of 80.8%.

## Conclusions

This pivotal randomised, blinded, positive controlled, multicentre, GCP-compliant, European pivotal field study demonstrated the effectiveness and safety of Credelio Plus, a combination of lotilaner at a dose rate of 20.0–41.5 mg/kg bodyweight and MO tablets at a dose rate of 0.75–1.56 mg/kg bodyweight administered orally to dogs naturally infected with *T. canis*,* A. caninum* and/or* T. vulpis*. A single oral dose of Credelio Plus was accepted free choice by 82.3% of the treated dogs, resulting in a ≥ 97.2% reduction against all of the canine intestinal nematode species assessed in this field study. This new combination treatment option of lotilaner + MO (Credelio Plus) provides dogs flea and tick prevention and control, intestinal parasite treatment and control of *T. canis*,* A. caninum* and* T. vulpis* and heartworm prevention. Thus, it can be used to provide broad-spectrum parasite control according to the approved label for dogs and their pet owners. This will contribute to owner compliance and simplifies the treatment recommendations from global scientific groups to prevent or treat these parasites. Additionally, these treatment recommendations may have a positive impact by decreasing the transmission of important zoonotic parasites and tick- and flea-transmitted disease agents.

## Data Availability

The dataset on summarising and supporting the conclusions of this article are included within the article. Due to commercial confidentiality of the research, data not included in the manuscript can only be made available to bona fide researchers subject to a fully executed non-disclosure agreement.

## Abbreviations

AE: Adverse event
CP: Control product
ESCCAP: European Scientific Counsel Companion Animal Parasites
FEC: Faecal egg count
GCP: Good Clinical Practice
GM: Geometric mean
IP: Investigational product
MO: Milbemycin oxime
